# Host and structure-specific codon usage of G genotype (VP7) among group A rotaviruses

**DOI:** 10.3389/fvets.2024.1438243

**Published:** 2024-11-08

**Authors:** Ziwei Liu, Simiao Zhao, Xinshun Jin, Xiaobo Wen, Xuhua Ran

**Affiliations:** School of Tropical Agriculture and Forestry, Hainan University, Haikou, China

**Keywords:** Rotavirus A, VP7, codon usage, host specificity, mutation, selection description number

## Abstract

Rotavirus A (RVA) infects a relatively wide host range. Studying the evolutionary dynamics of viral genomes and the evolution of host adaptations can inform the development of epidemiological models of disease transmission. Moreover, comprehending the adaptive evolution of viruses in the host could provide insights into how viruses promote evolutionary advantages on a larger scale at host level. This study aims to determine whether host specificity in codon usage existed. We used the Clustal W function within MEGA X software to perform sequence alignment, followed by construction of a phylogenetic tree based on the maximum-likelihood method. Additionally, Codon W software and EMBOSS were utilized for analysis of codon usage bias index. We analyzed codon usage bias (CUB) of host-specific G genotype VP7 to elucidate the molecular-dynamic evolutionary pattern and reveal the adaptive evolution of VP7 at the host level. The CUB of RV VP7 exhibits significant difference between human and other species. This bias can be primarily attributed to natural selection. In addition, the β-barrel structural domain, which plays a crucial role in viral transmembrane entry into cells, demonstrates a stronger CUB. Our results provide novel insights into the evolutionary dynamics of RVs, cross-species transmission, and virus-host adaptation.

## 1 Introduction

The Rotavirus (RVs), a member of the Sedoreoviridae family, is responsible for causing acute enteritis in infants and young animals (1). Rotaviruses possess genomes consisting of 11 dsRNAs that encode structural viral proteins (VP1–4, VP6, and VP7) and nonstructural proteins (NSP1–NSP 5/6). Reports from the Lancet Glob Health show that 108,322 deaths occur due to RV infection alone (2).

Notably, some RV strains can spread across species between host species. Considering that segmented viruses have the potential to undergo different degrees of independent evolution of genes in different segments (3), we have further thought about their potential risks. We know, for example, that influenza is a segmented virus that can cross species to cause a global pandemic. These zoonosis may lead to pandemics after sufficient adaptive genetic evolution has accumulated in new species (4). Therefore, a more comprehensive characterization of evolution among RVs in diverse hosts can provide an evolutionary foundation for monitoring and predicting the dissemination of mutant RV strains.

Viruses employ host-adaptive evolutionary mechanisms to fulfill their replication needs. Moreover, this evolutionary process is not simply a unilateral adaptation on the virus’s part but accompanied by the negative evolution of the virus by host receptors. In recent years, researchers have gradually uncovered mutual evolutionary influences between hosts and viruses, leading to an ongoing arms race. While describing the evolutionary landscapes of host–virus arms races, there exists the problem of the balance of the contradictory evolution of viral adaptations (5). Viruses urgently need the genome to evolve positive selection in response to adapt host rapidly. However, due to the basic functional requirements of virion assembly and replication, the protein sequence encoded by the virus cannot adapt to the negative selection of the host to the greatest extent. This creates a situation where in addition to amino acid levels, the adaptive evolution of viruses evolutionary changes at the nucleotide level as well (6). Researchers have discovered that viruses employ a cryptic mutational evolutionary strategy known as “synonymous codon mutation”. In which the amino acid sequence remains unchanged while only the triplet codon is altered, thereby resolving the paradox of adaptive viral-host evolution (7).

Organism genome exhibit a distinct inclination toward specific codons used when encoding for the same amino acids, defined as codon usage bias (CUB). Analysis of CUB in viruses can unveil the evolutionary forces at work in viruses. Viruses encounter analogous evolutionary pressures akin as all organisms, encompassing base mutation and natural selection (8). Nonetheless, due to their high mutation rates and extensive population size fluctuations, RNA viruses encounter these forces in a totally distinct manner compared to cellular organisms. The evolutionary capacity of RNA virus populations is critical for establishing successful infections, determining tropism, and triggering pathogenesis. The entire RV genome is subject to CUB (9). The predominant coat protein VP7, which determines the G genotype, engages in cellular receptor binding through its interaction with VP6 during RV entry into host cells. VP7 does not exert any influence on the synthesis of other viral proteins (10); however it plays a crucial role in mediating interactions with host cell and facilitating RV assembly by displacing the transient membrane through specific VP7 transmembrane structural domains (11), such as the β-barrel domain (12). By studying the codon usage of RV VP7 sequence, the adaptation evolution of RV VP7 to host was further elucidated.

In this study, we conducted an analysis of 172 RVA VP7 sequences from human, porcine, bovine, and bat, which are registered in the GenBank database. We confirmed the host variation in codon usage of RV VP7 and explored its molecular evolution. Our results can provide new perspectives on RV evolution, cross-species transmission, and virus–host adaptation, as well as provide a theory-driven strategy for the development of highly efficient antiviral vaccines.

## 2 Materials and methods

### 2.1 Strain selection

We retrieved complete nucleotide (nt) sequences of RVA VP7 for different species from GenBank. From the RVA VP7 sequences downloaded between 2015 and 2022, we randomly selected 172 for analysis, of which 51 were from human, 52 from bovine, 46 from porcine, and 23 from bat.

### 2.2 Experimental tools and parameters

Using Recombination Detection Program (RDP) version 5.0,^1^ we performed a recombination assessment of selected sequences to eliminate the effects of genetic-recombination events on subsequent phylogenetic-tree construction and CUB analysis. Recombination in VP7 sequences was assessed using seven methods: RDP, Bootscan, Maxchi, GeneConv, Chimera, Siscan, and 3 Seq. Sequences were considered recombinant if more than four out of the seven methods indicated recombination markers. Subsequently, potentially recombinant sequences were excluded in a followup experiment.

### 2.3 Sequence alignment and phylogenetic-tree construction

We used the Clustal W function within MEGA X software^2^ to align the selected sequences. Subsequently, we employed MEGA X software to construct a phylogenetic tree based on the maximum-likelihood method, with a bootstrap value of 1,000 replicates. The nucleic acid substitution model TN93 + G + I was selected, along with a confidence interval (CI) of 95%, while other parameters were set as default values for computation.

### 2.4 Codon usage bias index and analytical method

We used Codon W software^3^ and the cusp module in the European Molecular Biology Open Software Suite (EMBOSS)^4^ to analyze codon base data; effective number of codons (ENC) of the VP7 sequence; guanine-cytosine (GC) content of codon positions 1, 2, and 3 (GC1, GC2, and GC3); base content of codon position 3 (A3S, T3S, G3S, and C3S); GC content of synonymous codon position 3 (GC3S); as well as the frequency of codon usage.

#### 2.4.1 ENC and ENC plot analysis

CUB, as represented by the Effective Number of Codons (ENC) value, refers to variation in how frequently various codons corresponding to various amino acids are used. ENC value ranges from 20 to 61, where 20 indicates that only one codon is used per amino acid, and 61 indicates that each codon is used equally. We performed ENC plot analysis using GC3 and ENC as the x- and y-axes, respectively, which is an effective way to visualize the CUB of genetic data. The standard curve:


$$\mathrm{ENCexp}=2+GC3s+29/\left[GC3s2+\left(1-GC3s\right)2\right]$$

indicates that CUB is entirely determined by mutations without selection pressure, i.e., CUB is entirely determined by nucleic acid sequence composition. Based on the dispersion of the scatter around the standard curve combined with the ENC ratio frequency:


$$\mathrm{ENCratio}=\left(\mathrm{ENCexp}-\mathrm{ENCobs}\right)/\mathrm{ENCexp}$$

we could determine the factors affecting CUB.

#### 2.4.2 Parity Rule 2 analysis

Parity Rule 2 (PR2) analysis calculates adenine-thymine (AT) bias (A3S/(A3S + T3S)) by A3S and T3S, and GC-bias (G3S/(G3S + C3S)) by G3S and C3S, using the correspondence between the two to analyze the effects of selection and mutation on codon usage patterns. This analysis showed whether the only factors affecting CUB were random mutations (when codon A3 = T3 and G3 = C3) or mutations and natural selection acting together (when codon A3 ≠ T3 and G3 ≠ C3).

#### 2.4.3 Neutrality plot analysis

GC12 represents the average GC content at the first and second codon positions (GC1 and GC2), whereas GC3 refers to GC content at the third codon position (GC3). We used GC3 and GC12 as the x- and y-axes, respectively, to draw neutral plots for correlation analysis. Changes in third-codon position usually do not cause changes in coding amino acids, and base mutations at position 3 are subject to less selection pressure. Therefore, to assess CUB, it is essential to study the base composition of base position 3. If the correlation between GC12 and GC3 is significant and the regression coefficient is close to 1, mutation is the primary influence; otherwise, GC12 is highly different from GC3, and natural selection is the primary influence.

### 2.5 RVA encodes the proportion of synonymous codons of the same protein

Using EMBOSS cusp, we analyzed the codon usage of the four host-derived RVA. Scatter plots were created using GraphPad Prism version 9 (GraphPad Software, Inc., San Diego, CA, USA) to explore patterns of synonymous CUB in different host-derived RVA.

### 2.6 Table of codon usage for different host species

The codon usage of the host species was queried using the HIVE Codon Usage Tables (HIVE-CUTs) database.^5^ We used human (Taxonomy ID: 9606), porcine (Taxonomy ID: 9823), bovine (Taxonomy ID: 27592), and bat (Taxonomy ID: 9431) sequence data to generate the host species’ codon usage by HIVE-CUTs database.

### 2.7 Codon adaption index and relative codon deoptimization index

Codon adaption index, CAI, refers to the degree of alignment between the codon usage in the coding region and the optimal codon usage of the host cell. CAI values range from 0 to 1, with a higher value indicating a higher degree of similarity between the virus protein’s codon usage frequency and that of the host cell. This indicates that the more adaptive the virus is to the host cell, the higher protein expression. It calculates the CAI of the target gene relative to the host cell based on the reference sequence or frequency of codon usage of the known host cell. Relative codon deoptimization index, RCDI is used to assess the degree to which codon usage of a given gene is similar to that of the host cell and to test the level of deoptimization of the viral gene. RCDI values close to 1 indicate high similarity between viral and host genes, while RCDI values >1 indicate unoptimized codon usage patterns between viruses and their hosts.

We analyzed the CAI and RCDI values of VP7 genes for human, porcine, bovine, and bat species to explore the adaptation mechanism of the RVA VP7 gene relative to different hosts. The CAI and RCDI calculations were completed through online websites^6^ and RCDI calculations were completed through online websites (see text footnote 6).^7^

### 2.8 RVA VP7 three-dimensional structure drawing

We obtained the amino acid sequence of VP7 from the NCBI website. The 3D structure of VP7 was modeled from the amino acid sequence of RVA VP7 at the SWISS-MODEL website.^8^ From several predictive results given by the website, we chose the file with the highest homology, then used Chimera X software^9^ to download it and edit it according to the secondary structure of VP7.

### 2.9 Statistics and analysis

We used Microsoft Excel (Microsoft Corp., Redmond, WA, USA) to statistically collate RV VP7 information. And the GraphPad Prism 9 was used to conduct one-way ANOVA and two-factor ANOVA for the ENC values of the analysis series. Differences were considered statistically significant at *p* ≤ 0.05.

## 3 Results

### 3.1 Phylogeny of rotaviral VP7

**Figure 1:**
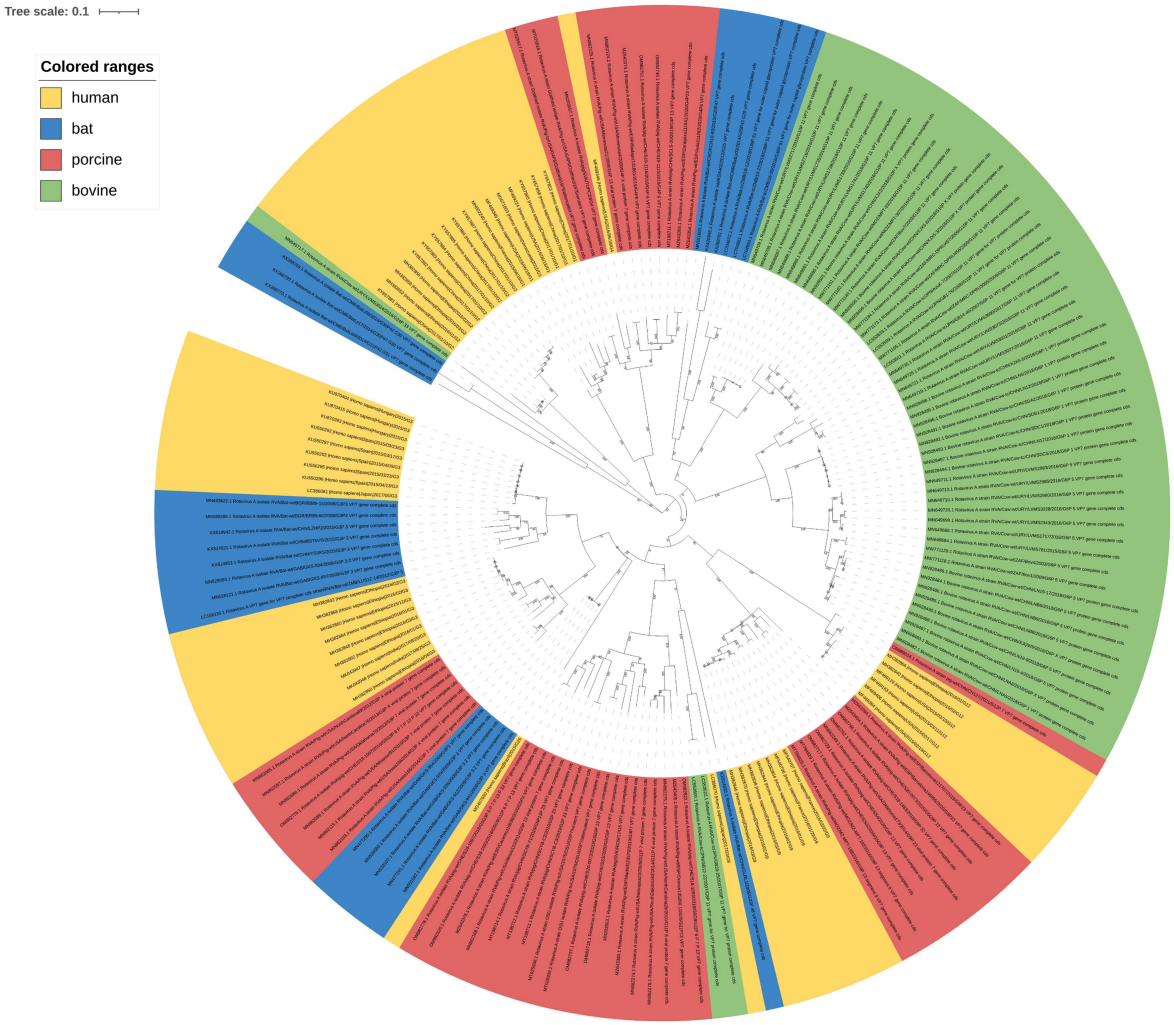
Phylogenetic-tree analysis of RVA VP7. The maximum-likelihood method was used to construct the phylogenetic tree. Human, porcine, bovine, and bat RVA VP7 sequences are indicated by yellow, pink, green, and blue, respectively; 100 × bootstrap is represented in the phylogenetic-tree branches.

The number of RVA G serotypes is increasing. And some rare serotypes have been isolated in humans, indicating a potential genetic variation trend for rotaviral VP7. A phylogenetic tree of RVA VP7 was constructed to evaluate the protein’s genetic evolution. We analyzed the complete sequences (Supplementary Table 1) of the downloaded fragments using RDP v5.0. No recombination events that could interfere with phylogenetic-tree construction were detected. Maximum likelihood (ML) phylogenetic-tree results (Figure 1) clustered into different branches based on RVA VP7 serotypes, with human- and porcine-derived RV VP7s intersecting each other in the phylogenetic tree, while bovine- derived RV VP7s in a single cluster. Bat-derived RVA VP7 serotypes appeared in multiple branches within the developmental tree. More importantly, we found that the phylogenetic results showed that the positions of VP7 sequences of the same serotypes in the phylogenetic tree clustered into different branches depending on the host. We further investigated codon usage patterns within these VP7 sequences to explore any potential relationship between codon usage and host specificity.

### 3.2 Effective number of codons of 172 sequences from four species

**Figure 2:**
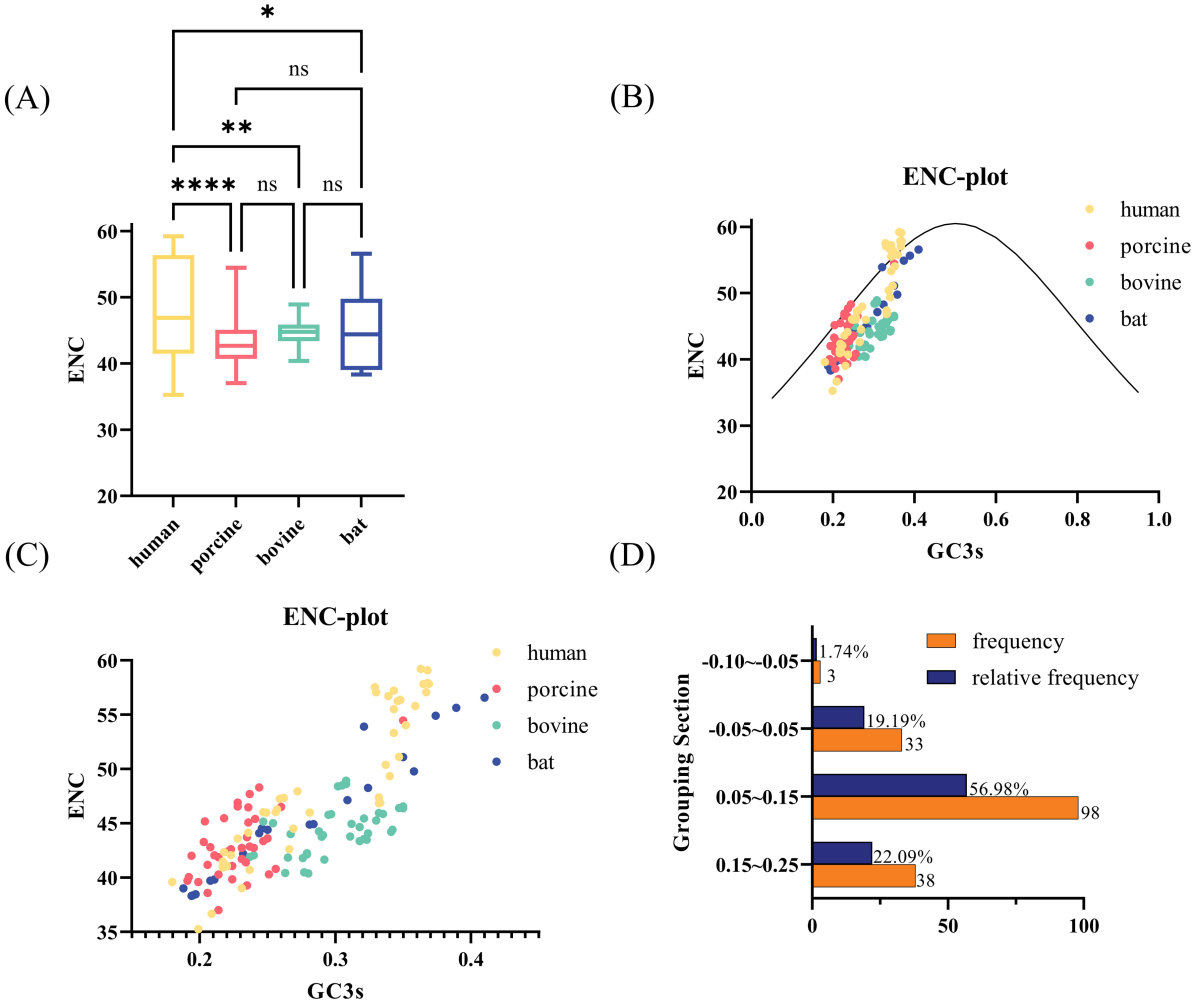
ENC-related analysis of RVA VP7. (A) Box line plot of ENC values for each strain from different hosts. Human, porcine, bovine, and bat RVA VP7 sequences are indicated by yellow, pink, green, and blue, respectively. (B) ENC plot of RVA, representing the relationship between ENC values and GC3 for each virulent strain. The solid curve plots the relationship between GC3s and ENC in the absence of selection pressure. ENC and GC3 scatters for most strains are evenly distributed below the standard curve. (C) Local zoomed in view of (B). Each scatter indicates ENC plot analysis results for the RVA strain. Yellow, pink, green, and blue scatter points indicate human, porcine, bovine, and bat RVA VP7 sequences, respectively. (D) Frequency distribution of ENC ratios for 172 sequences. ENC ratio was calculated as (ENC standard value − ENC value)/ENC standard value.

We calculated ENC values for each VP7 sequence (Figure 2). The ENC value ranges were 35.27–59.22 for human-derived RV VP7s (hRVA; Supplementary Table 1), 37.02–54.46 for porcine-derived RV VP7s, 40.39–48.93 for bovine-derived RV VP7s, and 38.32–56.57 for bat-derived RV VP7s (Figure 2A). ENC values for all VP7 sequence exceeded the threshold of 35, with a mean value of 45.35, indicating that the VP7 gene had weak overall CUB. Notably, ENC values of VP7 differed significantly between host humans and other species. ENC values were statistically different but not significant among other species. We employed ENC-plot (Figure 2B) to analyze factors affecting VP7 CUB, with most points scattering below the standard curve. Furthermore, by enlarging Figure 2B, it was observed that some human-, porcine- and bat-derived VP7 sequences were closer to the standard curve, while others tended to move downward and away from the curve (Figure 2C). The ENC ratio aptly demonstrates the disparities between the ENC values for VP7 sequences and the benchmark values. We calculated the ENC ratio of all sequences and grouped the 172 sequences into groups for analysis based on the ratio (Figure 2D). We further found that the ENC ratios of 33 sequences, including human- and bat-derived sequences, were distributed in the −0.05 to 0.05 interval. This result is close to the standard curve, indicating that mutations mainly influenced the preferences of these 33 sequences. Except for the above-mentioned 33 sequences, ENC values of sequences were located further from the standard curve, indicating that VP7 CUB of these sequences was more influenced by natural selection.

### 3.3 Parity Rule 2 analysis and neutral analysis of 172 sequences from four species

**Figure 3:**
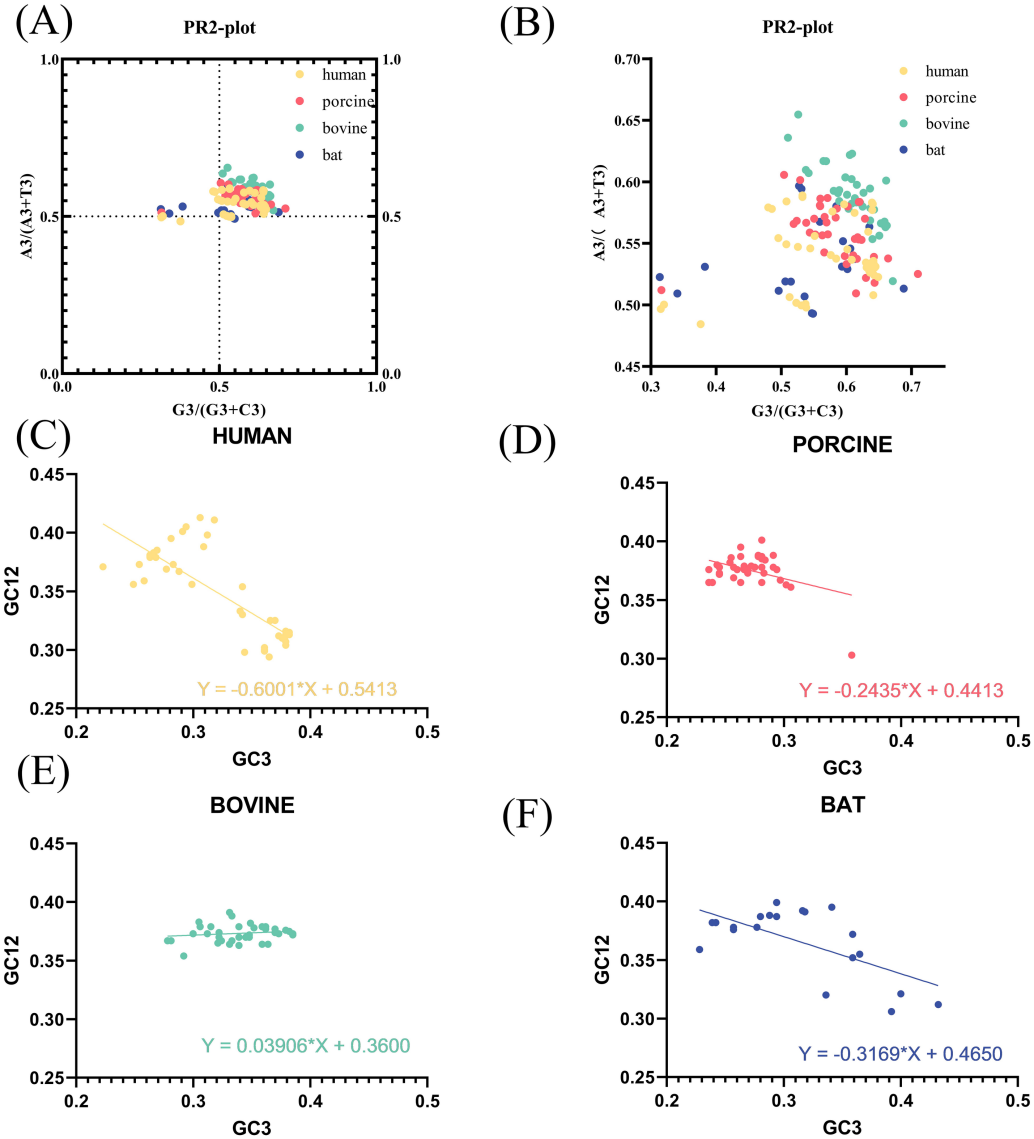
PR2 plot and neutral plot analyses of RVA VP7. (A) PR2 plot analysis of sequences of different host-coding areas of RVA. In the PR2 scatter plots, the points of all RVA strains are distributed in the upper-right quadrant and are relatively concentrated. (B) Local magnification of (A). (C–F) Neutral plot analysis of RVA VP7 (GC12 vs. GC3). The straight line represents the regression line; the regression equation is also shown. Slopes significantly deviating from 1 show evidence of natural selection, while slopes near 1 suggest mutational selection is the driving force of codon usage patterns.

Having determined that codons of the VP7 sequence had different codon bias due to host species, we performed PR2 and neutral analyses of VP7 sequence to determine further factors affecting VP7 CUB. PR2 plot results (Figure 3A) showed that for most sequences, points were scattered in the upper-right quadrant. Furthermore, by enlarging Figure 3A, we can see that most sequences had the ratio of A3/(A3 + T3) to G3/(G3 + C3) greater than 0.5, except for a few human- and bat-derived VP7 sequences (Figure 3B). In other words, the composition of the third codon base in the triplet codon of the RVA VP7 coding region was A > T and G > C. Purines were used more frequently than pyrimidines, further suggesting that base mutation and natural selection had some influence on RVA VP7 codon usage preference. The neutrality plot (Figures 3C–F) shows that the value ranges of GC12 and GC3 were, respectively, 0.29–0.41 and 0.22–0.43, and the regression coefficient (i.e., slope) of VP7 was 0.3120. Meanwhile, the correlation coefficient (r) of GC12 and GC3 was 0.3269, which was not significant. It could be tentatively determined that natural selection was one of the main factors affecting the preference for RVA VP7 codon usage. In a comparison of different hosts, regression coefficients were 0.6001 for human-derived VP7 (Figure 3C), 0.2435 in porcine-derived VP7 (Figure 3D), 0.03906 in bovine-derived VP7 (Figure 3E), and 0.3169 in bat-derived VP7 (Figure 3F). Among the regression coefficient of the human-derived VP7 was more significant than 0.5, indicating its influence also by the specific mutations.

### 3.4 Frequency of synonymous codon usage

**Figure 4:**
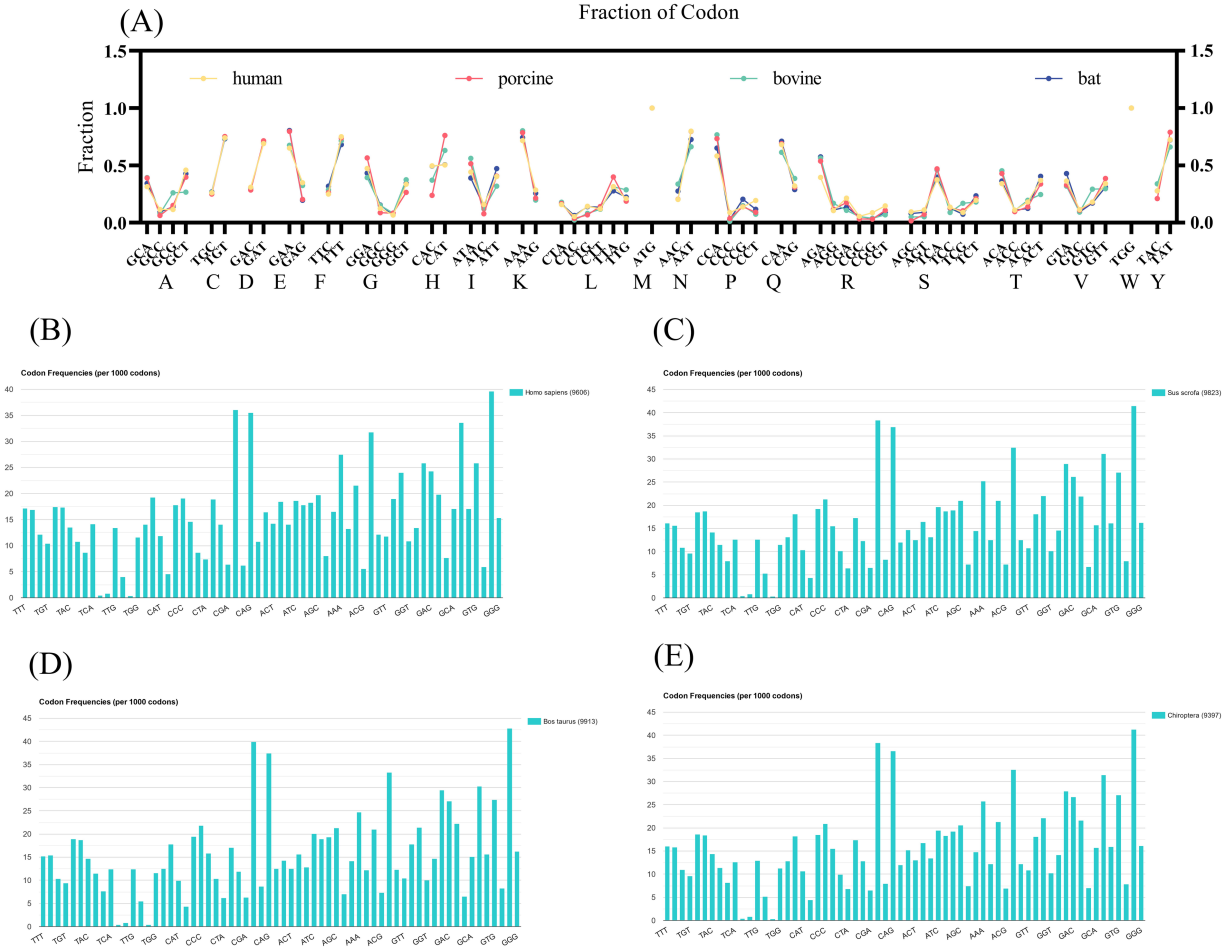
Host synonymous codon usage and frequency of RV VP7 synonymous codon usage patterns. (A) Quantitative indicators of changes in amino acid synonymous codon usage patterns in different RVA hosts. Each scatter represents frequency of use of synonymous codons for each strain (yellow indicates human RVA VP7, pink indicates porcine RVA VP7, green indicates bovine RVA VP7, blue indicates bat RVA VP7). (B–E) The codon usage of the four host species. Represents the frequency of occurrence of amino acids per 1,000 triple codons. The names of the 20 amino acids are indicated by abbreviations.

To further validate our results, we probed the frequency of VP7 synonymous codon usage encoding the same amino acids in different hosts (Figure 4A). Histidine was the amino acid with the most significant differences. We compared the frequency of histidine-synonymous codon usage among the four host species. Frequencies of histidine, bat-derived and human-derived CAC, and CAT use were comparable. However, porcine- and bovine-derived CAT use was higher, at 0.762 for porcine and 0.63 for bovine. To explore the reasons for this difference, we queried the host species codon usage (Figures 4B–E). The frequency of the CAC codon was higher than that of the CAT codon in the human genome and hRV VP7, which might be due to codon adaptation of the virus to the host.

### 3.5 CAI and RCDI assessments

**Figure 5:**
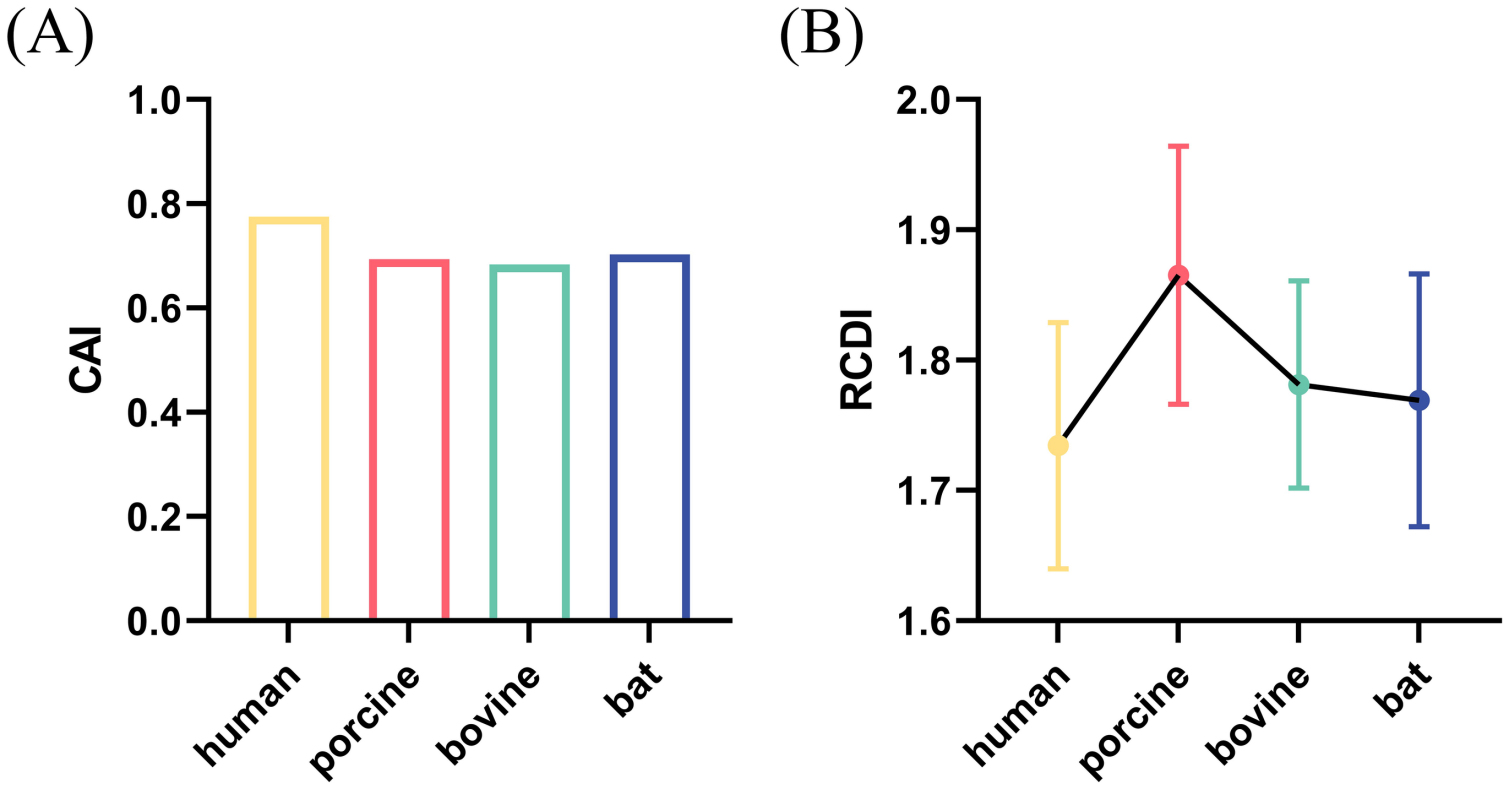
CAI value (A) and RCDI (B) value of VP7 gene for four species: human, porcine, bovine, and bat.

To assess the adaptation of the RVA VP7 CUB to its host, CAI analysis was performed. The results of our analysis showed that CAI values of RVA VP7 in human, porcine, bovine and bat expression systems were 0.775, 0.694, 0.684, and 0.703, respectively (Figure 5A). By comparison, the VP7 gene showed the highest CAI value relative to human indicating that it had the best adaptability. It is worth noting that the VP7 gene had the lowest adaptability relative to bovine. We analyzed the RCDI to further explore the adaptability of RVA VP7 to various hosts (Figure 5B). Of the four viruses of different host origin, the RCDI value of human RVA VP7 was the closest to 1 and the codon deoptimization was the greatest.

### 3.6 Deviations in codon usage for different structural domains of VP7

**Figure 6:**
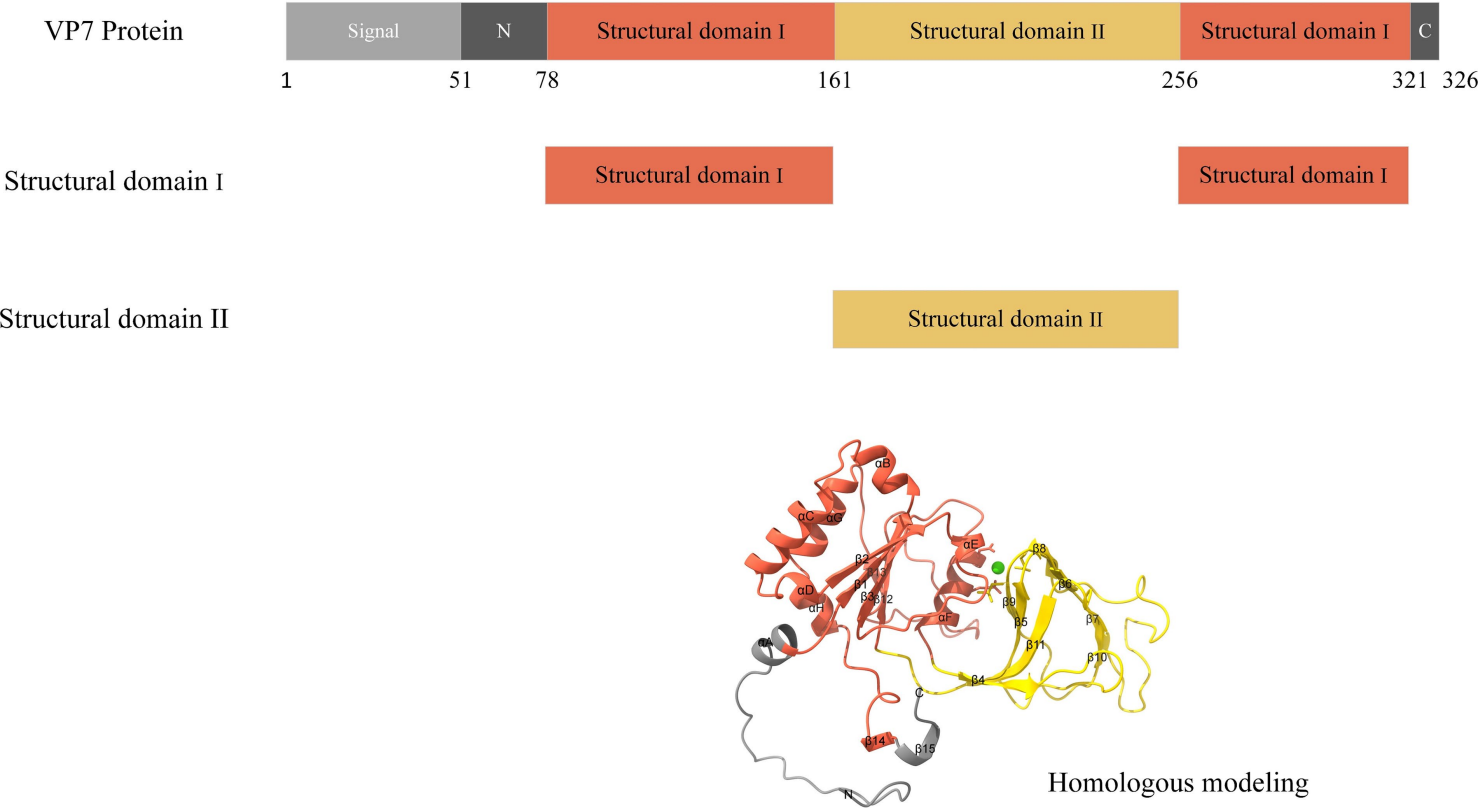
Schematic of the structure of RVA VP7. Light gray regions 1–51 is the signal sequence. Dark gray regions 51–78 and 321–326 are the disordered N- and C-terminal arms, respectively. Red regions 78–161 and 256–321 are structural domain I; yellow regions 161–256 are structural domain II.

**Figure 7:**
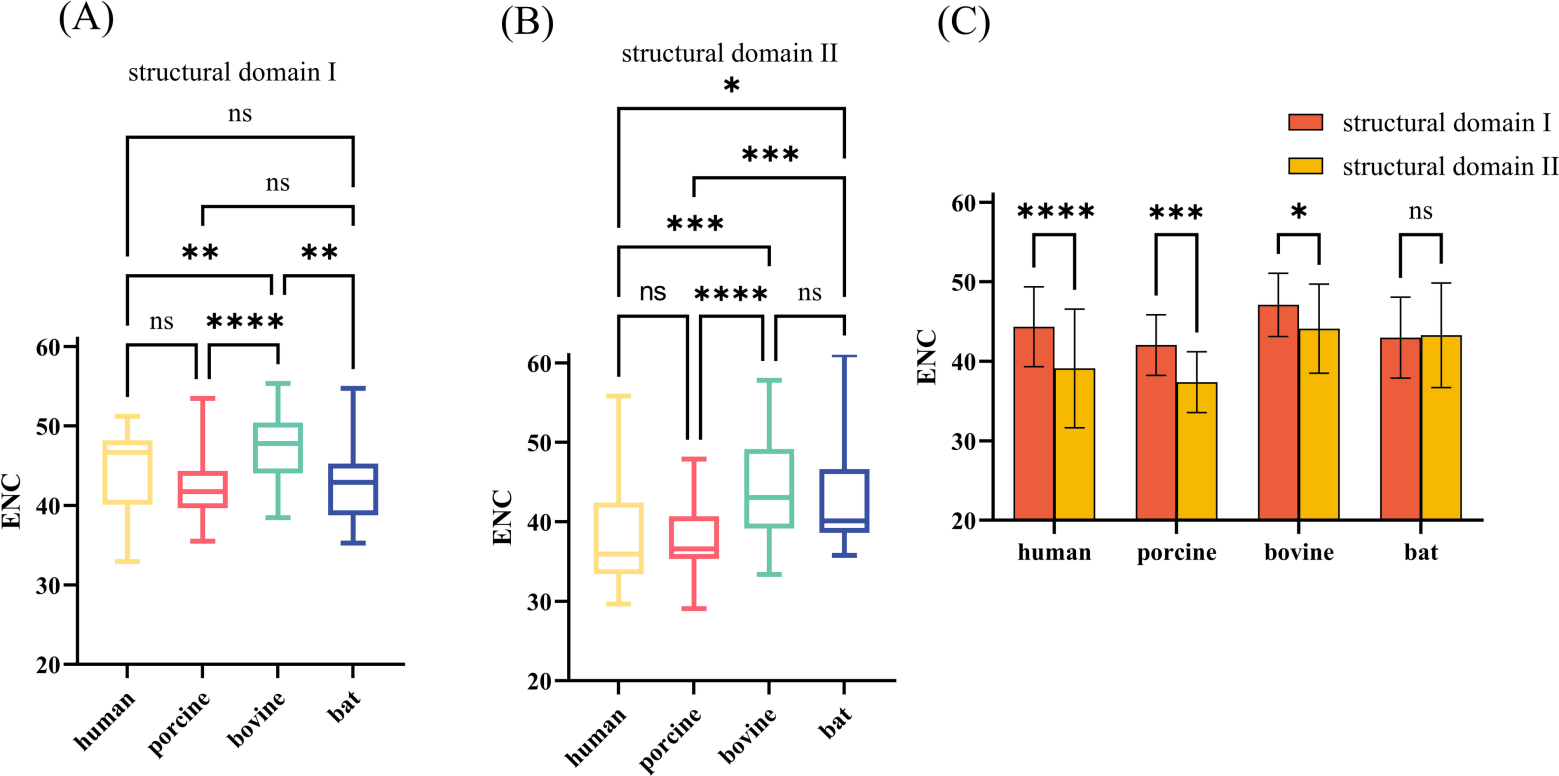
ENC values of nucleotide (nt) sequences corresponding to different structural domains of RVA VP7. (A,B) These two plots compare ENC values of nt sequences corresponding to structural domains I and II of different hosts. (C) Comparison of ENC values of corresponding nt sequences of different structural domains of the same host.

The VP7 of RV is about 326 amino acids long and contains two compact structural domains based on its steric VP7 structure. Amino acid sequences 78–161 and 256–321 comprise structural domain I, a “Rossman fold,” and structural domain II is encoded by amino acids 161–256, with a jelly roll beta-sandwich inserted between α-helix D and β-strand 11 (Figure 6). We combined the base sequences of structural domain I for CUB analysis and found that CUB also differed significantly at different positions in the same gene (Figures 7A,B), although there was no pattern to this difference. Structural domain II had a strong CUB (Figure 7C).

## 4 Discussion

Selection of base sequences with identical amino acids for codon analysis has profound implications for studying the molecular evolution of genes. Among the 172 gene sequences we analyzed, codon usage bias was generally low and the degree of codon preference for VP7 sequences differed significantly across hosts. In a subsequent analysis of the causes of codon preference, it was found that base mutations lead to codon preference, but natural selection is the main cause of VP7 codon preference. This study clarifies that rotavirus VP7 gene codons usage is host-specific and adaptive. Furthermore, it was observed that the β-barrel structural domain, which plays a crucial role in viral transmembrane entry into cells, exhibited stronger Codon Usage Bias (CUB).

The low overall codon usage bias of RV VP7 may be attributed to the fact that RNA viruses are unable to synthesize tRNA. They need to synthesize their own proteins through the host’s translation machinery and resources (13). Hence viruses may select specific synonymous codons as a means of controlling viral protein translation, allowing them to infect their hosts and spread through host populations with varying efficiencies. Synonymous codons in viral coding sequences are important mediators of viral adaptation to the host (14). It has been shown that the low codon bias of RNA viruses is an advantage for their efficient replication in host cells, which reduces the competition between the virus and the host for the synthesis machinery (15). The high ENC values of RV VP7 indicate that all codons are used almost without preference, and a similar situation has been documented in other RNA viruses, such as the infectious gastroenteritis virus (16). RNA viruses through this low codon bias permits efficient replication in host cells.

Viral adaptation to the host in codon usage is a long-term evolutionary feedback mechanism. So if RV strains have different hosts, the differences in preference for the VP7 gene are not yet known. Our study found that there are differences in codon usage for the same gene because of different hosts. In our results, we showed that there were significant differences in the degree of codon bias between human and other derived rotavirus VP7 genes. Furthermore, our findings indicate that although base mutations do influence RV VP7 codon preference, natural selection plays a more prominent role in shaping this bias. Each organism is characterized by its specific codon usage, reflecting the evolutionary forces acting on its genome. Early findings suggest that mutational bias is the primary driver of codon bias (17), but natural selection also plays a role in shaping genomic codon usage (18). Among various drivers of natural selection, translational selection or adaptation of tRNA libraries to ensure translation efficiency and accuracy has been proposed in many organisms from prokaryotes to eukaryotes (19). Notably for viruses like RVs which cannot synthesize tRNAs themselves, translational selection requires adapting their codon usage to their host cell DNA library (20). This elucidates why natural selection plays a dominant role in the drivers of codon bias in RV VP7. Not coincidentally, other multihost viruses such as Crimean–Congo hemorrhagic fever virus (21) and Zika virus (22) has also been found to have a combination of natural selection and mutational pressure leading to CUB.

By contrast, the codon usage of the bat-derived VP7 was not significantly different from that of other hosts, which might be because infected bats are tolerant of infection. Infected bats, acting as special viral reservoirs (23), play important roles in the ecosystem of the pathogen and recipient host species (24). Therefore these viruses do not require adaptive evolution to infect bats. In addition, these viruses can spill over undetected. Infected bats serve as natural viral reservoirs, resulting in close genetic relatedness between bat strains and those found in other hosts. This might explain why bat-derived VP7 is distributed across multiple branches within the phylogenetic tree. Another contributing factor could be that bats, as the only flying mammals, produce substantial amounts of reactive oxygen species (ROS) and limit oxidative stress (OS) via modulated genes (25), which might lead to reduced viral replication and morbidity (26). Reduced viral replication might have forced the RV genome in bats to evolve adaptively with a decreased positive selection pressure. Therefore, ENC values of the VP7 sequence of the bat strain do not differ significantly from those of the other species.

In our subsequent study, we further examined our conjecture. We conducted a comparative analysis of RV VP7 codon usage frequencies among its respective host species. Notably, histidine emerged as the amino acid exhibiting the most significant disparity in RV VP7 utilization across the four host species. Histidine is encoded by either CAT or CAC, with CAT being utilized at frequencies of 0.504, 0.762, 0.630, and 0.505 in human, porcine, bovine, and bat, respectively. Interestingly, while CAC was predominantly favored in host cells for encoding histidine, the lowest frequency of VP7 CAT usage in the human strain may be an evolutionary adaptation of the virus to the host codon. It is worth mentioning that recent literature has extensively demonstrated that viral codon usage is adaptive to their respective host, furthermore highlighting numerous similarities between viruses and hosts regarding their codon utilization patterns. For example, the Usutu virus has overlapping antagonistic codons across hosts, enabling more efficient replication in highly adapted hosts and stable survival and transmission in some low adapted hosts (27).

The adaptive relationship between RV VP7 and host was further analyzed. Our results show that hRV is the most adaptable in its host, which is consistent with RCDI results. The high prevalence of hRV can also confirm our results. In addition, a lower level of adaptation to the natural host than to the terminal host may contribute to the long-term coexistence and circulation of the virus in the natural host (28). Therefore, although RV is first found in children with diarrhea, it may not be the natural host of RV. However, the level of adaptation needs further research to confirm, especially in wet experiments.

The frequency of synonymous CUB has been demonstrated to differ between host species; however, whether differences are triggered within genes by location and function is unknown. The VP7 glycoprotein subunit core folds into two compact structural domains. Structural domain I adopts a “Rossmann fold,” and a jelly roll beta-sandwich (domain II) inserted between the α-helix D and β-strand (12). Structural domain II exhibits a β-barrel structure that plays a complete and necessary role in bacterial outer membrane protein membrane spanning (29). RVs infect cells by directly penetrating the cell membrane (30), which we hypothesize might be related to the β-barrel structure. Additionally, Structural domain II contains two calcium binding sites in RVs. More importantly, calcium ions play crucial roles in RV entry, transcriptional activation, morphogenesis, cell lysis, virion release (31, 32). During RV entry into the host cell, the three dimeric structures of VP7 are stabilized by Ca^2+^ (33) while displacing the transient membrane to assemble the RV (11). We calculated the ENC values of the two structural domains and found host specific differences in their codon usage patterns. As mentioned earlier, structural domain II had higher CUB, which we speculated to be necessary for entering and replicating within the host cell.

Analysis of CUB can be used to explore the development of RV vaccine combinations through codon deoptimization. Deoptimized viral genomes use less abundant or rare transfer RNAs (tRNAs) during replication in the host cell, resulting in lower viral translation rates, which in turn leads to lower replicating viral fitness and higher immune responses (34). By contrast, deoptimized rescued viruses contain multiple mutations and pose challenges for restoration to their original virulence (35). Poliovirus (36) and enterovirus A71 (37) have been rescued using codon deoptimization to produce attenuated strains with good immunogenicity. This strategy holds promise for the development of safe, stable, and protective attenuated vaccine strains. Recent advancements in constructing a plasmid based reverse genetic system for RVs have facilitated the rescue of attenuated RVs or generation of an improved vaccine backbone based on host codon usage preferences (38).

One must note that a majority of the rotavirus sequences originate from products sequenced post-isolation in cellular culture, as opposed to direct sequencing of clinical isolates. Given that diverse cell lines such as MA104, Caco-2, etc., are utilized for isolation, it is uncertain whether the strains that have not be isolated exhibit superior codon bias and host adaptation. Anyway, this study revealed that codon usage of rotavirus VP7 sequences was host-specific. The combination of natural selection and base drift shaped the codon usage of the RV VP7 gene. The role of natural selection is more prominent. We found that codon usage patterns of different functional and structural domains of the same gene were differential and host-specific. These findings provide valuable insights into the molecular evolution and protein expression strategies employed by RVs.

## Data Availability

The original contributions presented in the study are included in the article/Supplementary material, further inquiries can be directed to the corresponding authors.

## Footnotes

^1^http://web.cbio.uct.ac.za/~darren/rdp.html

^2^https://www.megasoftware.net/citationsto

^3^https://codonw.sourceforge.net/

^4^https://www.bioinformatics.nl/cgibin/emboss/help/cusp

^5^https://dnahive.fda.gov/, accessed April 6, 2023.

^6^http://genomes.urv.es/CAIcal/E-CAI/

^7^http://genomes.urv.cat/CAIcal/RCDI/

^8^https://swissmodel.expasy.org/

^9^https://www.cgl.ucsf.edu/chimerax/
